# Mentorship among healthcare researchers: a social network analysis

**DOI:** 10.3389/frhs.2025.1514379

**Published:** 2025-07-17

**Authors:** Bo Kim, Erin A. Pleasants, Jennifer L. Sullivan, Amy M. Linsky

**Affiliations:** ^1^ Center for Health Optimization and Implementation Research (CHOIR), VA Boston Healthcare System, Boston, MA, United States; ^2^ Harvard Medical School, Boston, MA, United States; ^3^ Transformative Health Systems Research to Improve Veteran Equity and Independence Center of Innovation (THRIVE COIN), VA Providence Healthcare System, Providence, RI, United States; ^4^ Brown University School of Public Health, Providence, RI, United States; ^5^ Boston University Chobanian & Avedisian School of Medicine, Boston, MA, United States; ^6^ New England Geriatric Research Education and Clinical Center, VA Boston Healthcare System, Boston, MA, United States

**Keywords:** mentorship, research career, healthcare workforce, social network analysis, workplace relationships

## Abstract

**Introduction:**

Mentorship is an active workplace relationship between a mentor and a mentee, aimed at mutual career advancement, which is vital for both employee growth and organizational success. To improve their mentorship structures and processes, organizations must first assess their current practices. Thus, we developed and conducted a cross-sectional survey to evaluate mentorship among employees at a two-site federally funded health services research center.

**Methods:**

We surveyed Center investigators and other employees (henceforth “staff”), gathering data on mentors, mentees, mentoring relationships, and satisfaction with the Center's mentoring infrastructure. We used social network analysis to examine both formal and informal mentoring relationships and assessed the association of employee connectedness in these networks with reported satisfaction.

**Results:**

There were 120 respondents (62.2% response rate). A greater percentage of investigators, compared to staff, had at least one formal mentor (55.8% vs. 25.0%) and one formal mentee (57.7% vs. 10.3%), and investigators had more informal mentors within the Center than staff (4.94 vs. 3.59, *p* = 0.0485). Investigators reported higher satisfaction with mentorship compared to staff (6.63 vs. 5.25, *p* = 0.002) and had more formal mentoring relationships with other investigators than staff had with other staff (0.06 vs. 0.01 degree centrality, *p* < 0.0001). Combining formal and informal mentorship across both investigators and staff, compared to formal mentorship alone, showed fewer degrees of separation (1.32 vs. 3.41 mean distance, *p* < 0.0001). For the combined formal and informal mentorship network across both investigators and staff, satisfaction with mentoring was associated with having more connections with network members who were connected with each other (*r* = 0.998, *p* < 0.0001).

**Discussion:**

To foster connections among employees, research organizations may create opportunities for open communication and collaborative problem-solving. Our survey and findings are timely given the growing emphasis on mentorship's importance for successful careers, motivated employees, and workplace productivity.

## 1 Introduction

Mentorship is defined as a dynamic and reciprocal relationship in a work environment between a mentor and a mentee, aimed at promoting the career growth of both (1). Models of career success emphasize mentoring as a key element that impacts satisfaction for both mentor and mentee (2, 3). Notably, these models depict mentoring as an organizational effort, one with structures, processes, and norms as defined and practiced by the employees of the organization.

Research organizations increasingly recognize the importance of mentoring, not only for employees' career development but for organizational success. Mentoring influences employees' career trajectories (4) as well as work satisfaction, which can directly affect their productivity (5). As a result, successful mentoring can increase an organization's achievements. Research mentorship has been found to be pivotal in enhancing the competencies of healthcare professionals (and in turn, their organizations) in evidence-based practice and research methodologies, providing not only professional development but also emotional support that boosts self-confidence and skill acquisition among mentees (6, 7). For mentors, mentoring relationships are shown to increase their career success and satisfaction (8). This high value of mentorship is well recognized by many research funding agencies, exemplified by their requirement that applying organizations clearly demonstrate the ability to provide high-quality mentorship (9).

Organizations trying to improve their mentorship structures, processes, and norms must first accurately assess their current mentoring practices. Specifically, it is important to understand (i) existing mentoring relationships and how they are structured, (ii) factors that impact employees' perceptions of and participation in mentoring, and (iii) employee preferences for mentoring practices, both existent and aspirational. It is also important to recognize that mentorship may be formal or informal, where formal mentorship is a structured relationship in which an organization pairs a mentor and mentee as part of an official mentoring program, while informal mentorship arises naturally through shared interests and personal rapport between a mentor and mentee (10). However, there are no established methods to evaluate mentoring practices within a research organization. Research comes with its own specific nuances of necessary mentoring for academic scholarship that differ from other industries, such as navigating ethics review processes and grant writing, and thus it needs a tailored approach to evaluating its mentoring practices.

Furthermore, although some studies have examined mentorship structures beyond the traditional dyadic pairing between one mentor and one mentee (11, 12), they have conceptualized such non-dyadic structures primarily as representations of how specific mentees and/or mentors are related, rather than as representations of mentoring relationships within a research organization. This latter organization-level representation of employee relationships has been adopted by organizations targeting process improvements, including in the form of assessing employee social networks [i.e., sets of individuals and groups connected by similarities and their interactions with one another (13)] to leverage existing relationships for the improvement efforts (14). Even though it is recognized that mentorship can be viewed through this lens of who is connected to whom in a mentorship social network (15), we are unaware of social network analysis being applied to examine mentorship within research organizations.

Therefore, we developed and deployed a cross-sectional survey to assess mentorship among employees at a two-site federally funded research organization. We then used social network analysis to understand the organization's current mentoring relationships, practices, and attitudes. We outline below the steps we took and our findings, followed by a discussion of the potential utility of our methods for other research organizations seeking to assess and enhance their own mentoring practices.

## 2 Methods

### 2.1 Setting and participants

We surveyed all employees at a two-site federally funded health services research center (“the Center”) that is located in the northeast region of the United States and is embedded within a larger healthcare organization. The Center is affiliated with seven academic institutions, and its mission includes training the next generation of health services researchers. The Center's investigators typically have doctoral and/or professional level education (e.g., PhD, MD) and have academic affiliations with universities. Post-doctoral and physician fellows, because their fellowship involves them being mentored by the Center's investigators, were considered as investigators for analysis purposes. The Center's other employees (henceforth “staff”) include individuals who typically have bachelor or master level education (while some have doctoral degrees), and their roles include research assistant, project manager, data analyst, administrative specialist, and budget officer. Across both investigators and staff, the Center has approximately 190 employees.

### 2.2 Survey development

Two authors (AML and JLS) determined the domains of interest for the survey, considering the priorities and needs of the Center and its leadership. These domains included the prevalence of, barriers to, and satisfaction with formal and informal mentorship from the Center members' perspective. AML and JLS searched extant literature for other surveys related to mentorship, by using search strings in PubMed related to mentorship program, research center, and satisfaction survey, as well as their synonyms and variations. No surveys identified through this search comprehensively covered the determined domains of interest, so the initial survey draft for this project included *de novo* items for assessing mentoring relationships. The draft was circulated to Center leadership and other key members (e.g., the mentorship director, lead managers). In particular, recognizing the value of both formal (e.g., designated on a career development award proposal) and informal (e.g., between employees in similar career stages) mentorship, we included questions about both types of mentorship. We also included questions about the respondent's role, site, years at the organization, and career stage, to account for the Center's contextual circumstances at the time of the survey. As understood by the authors and by the Center's leadership, mentorship director, and lead managers, these circumstances were that the Center (i) had more formal mentorship structures in place for investigators than for staff, (ii) existed across two separate sites that differed in their setting, history, and norms, and (iii) consisted of employees at various career stages and with different numbers of years at the organization. After multiple iterations, the finalized survey consisted of 29 items.

### 2.3 Survey content

**Figure 1:**
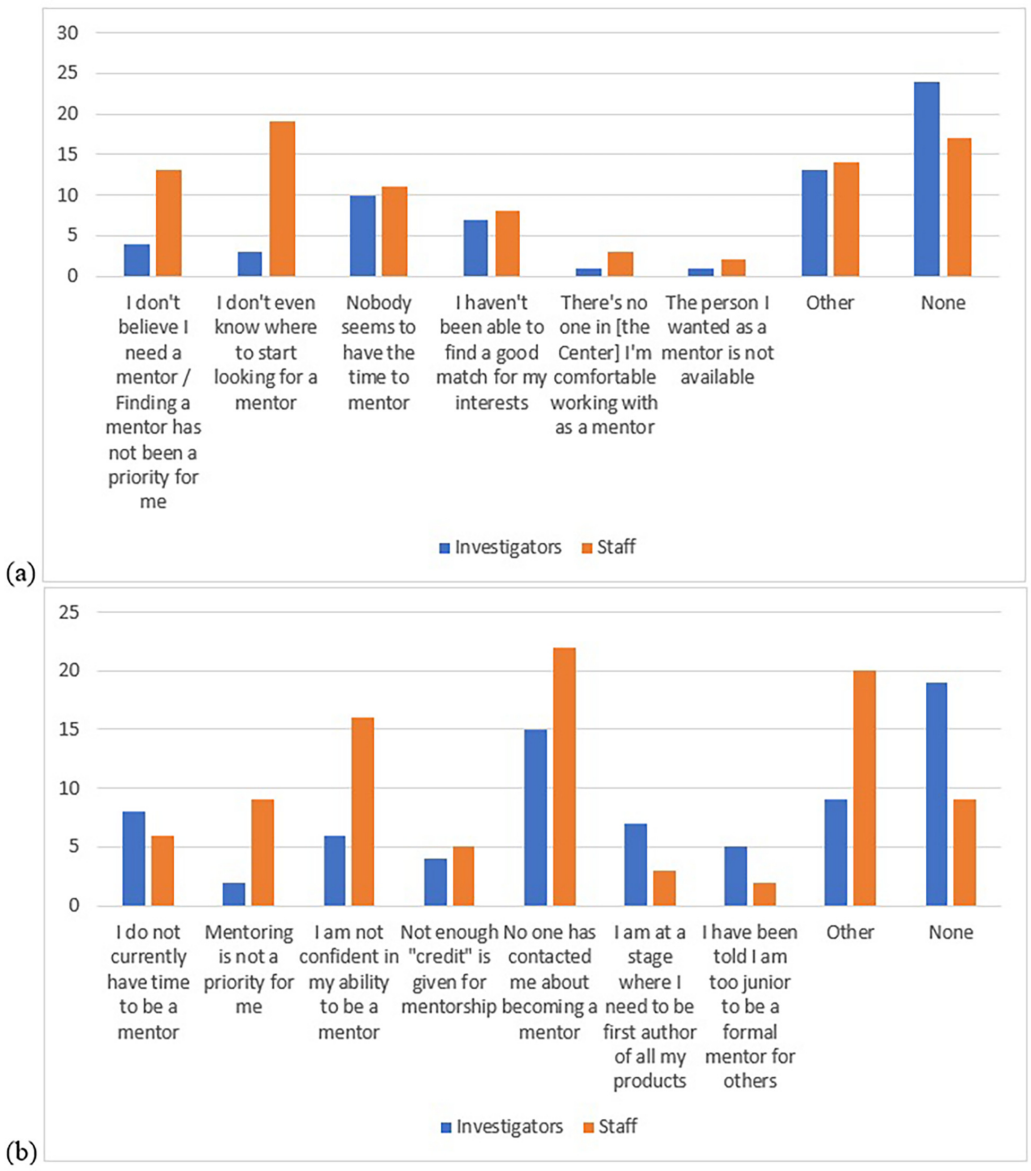
Limiting factors for **(a)** finding a formal mentor and **(b)** becoming a formal mentor; frequences compared between investigators and staff.

The first part of the survey elicited information on respondent characteristics (e.g., role, site, years at the organization, career stage) and the number of formal mentors, formal mentees, and informal mentors internal and external to the Center. For each of the questions asking about the number of mentors or mentees, respondents could select (i.e., designate) up to 10 individuals, either from a list of all Center employees or by writing in mentors/mentees from outside of the Center. To inform improvement efforts, the survey then used a checklist format to ask about barriers to mentorship—one item each on finding a formal mentor and becoming a formal mentor. (The checklist items are shown in Figure 1 along the horizontal axis of the charts.) The survey concluded with an item about satisfaction with the current state of mentorship at the Center, using a 10-point Likert scale (1 = “not satisfied at all” to 10 = “very satisfied”).

### 2.4 Data collection

Center leadership introduced the survey to the employees at Center-wide meetings and encouraged their participation. All employees at both sites were emailed an invitation to participate, along with a link to complete the survey. Non-responders were sent up to two reminders at one-week intervals. Survey data were collected and managed using the web-based REDCap (Research Electronic Data Capture) survey application.

### 2.5 Data analysis

Using SAS 9.4 (SAS Institute, Cary, NC), we ran frequencies and percentages to describe characteristics of respondents (i.e., role, site, years at the organization, and career stage) and responses to items about the number of mentors or mentees and overall satisfaction with the Center's mentoring infrastructure. To compare mean numbers of mentors or mentees and mean satisfaction across respondent characteristics, we used two-sample *t*-tests and analyses of variance for comparing two and more than two groups, respectively. For barriers to mentorship, we identified the most common responses by investigators and staff.

To further examine the formal and informal mentoring relationships among investigators and staff, we applied social network analysis [i.e., use of network and graph theory to investigate social structures (16, 17)] to survey data in which respondents designated specific individuals as their formal mentors, formal mentees, and informal mentors. We considered four directed networks: (1) formal mentorship among investigators, (2) formal mentorship among staff, (3) formal mentorship among investigators and staff, and (4) formal and informal mentorship among investigators and staff. For each network, we generated a visual network map; nodes represent survey respondents (Network 1 investigators only, Network 2 staff only, and Networks 3 and 4 investigators and staff), as well as individuals who did not respond to the survey but were designated by one or more respondents as a mentor or mentee (Networks 1–3 formal only, Network 4 formal and informal). We also computed the following social network analysis metrics:

• Network density: Proportion of connections between the network members among all possible connections between the network members
• Mean distance: Average number of steps between an individual and other network members, where a step is a direct connection between two individuals (e.g.; if Individual X designates Individual Y, then the number of steps between Individuals X and Y is 1; if Individual X additionally designates Individual Z, yet Individuals Y and Z do not designate one another, then the number of steps between Individuals Y and Z is 2)
• Clustering coefficient: Proportion of connections that exist among the other network members to whom an individual is connected, compared to all possible connections among those other members
• Degree centrality: Proportion of other network members either designating or designated by an individual among all other network members
• Betweenness centrality: Inverse of the average number [i.e., 1/(average number)] of steps between an individual and other network members (this value is higher for individuals who have fewer steps between themselves and other network members)

For mapping and computations, we used the sna package of R statistical software (v4.1.2) and SocNetV (Social Network Analysis and Visualization Software).

For the network density metric, we used a chi-square test to compare the proportions between Networks 1 (investigators, formal mentorship) and 2 (staff, formal mentorship). For Networks 3 (investigators and staff, formal mentorship) and 4 (investigators and staff, formal and informal mentorship), we used a McNemar's test to compare the proportions. For the other metrics, we used two-sample *t*-tests to compare their mean values between Networks 1 and 2. For Networks 3 and 4, we used paired-sample *t*-tests to compare their mean values and examined the correlation of metrics with satisfaction with the Center's mentoring infrastructure.

### 2.6 Project ethics determination

This project was conducted as a quality improvement activity for the Veterans Health Administration and was deemed by the Research & Development Committee at the VA Boston Healthcare System (Boston, Massachusetts, USA) not to be research; therefore, it was not subject to review by the Institutional Review Board.

## 3 Results

Of the 193 investigators and staff invited to participate, 120 (62.2%) responded to the survey. Table 1 summarizes respondent characteristics. Fifty-two (43.3%) and 68 (56.7%) respondents were investigators and staff, respectively, and 83 (69.2%) respondents had been at the organization for less than 10 years.

**Table 1:** Respondent characteristics.

Characteristic	n (% of *N* = 120)
Role at the center	
Investigator	52 (43.3)
Staff	68 (56.7)
Site	
Site A	54 (45.0)
Site B	66 (55.0)
Years at the organization	
0–3	50 (41.7)
4–9	33 (27.5)
10–19	28 (23.3)
20 or more	9 (7.5)
Career stage	
Early-career investigator	32 (26.7)
Mid-career investigator	12 (10.0)
Late-career investigator	8 (6.7)
Staff	68 (56.7)

### 3.1 Formal and informal mentorship

Overall, a majority of investigators had at least one formal mentor (55.8%), at least one formal mentee (57.7%), and at least one informal mentor (59.6%). In contrast, a minority of staff reported at least one formal mentor (25.0%), at least one formal mentee (10.3%), and at least one informal mentor (42.6%). Table 2 compares the mean numbers of mentors or mentees across respondent characteristics. For formal mentorship, the mean number of formal mentees was significantly different for respondents who (i) had been at the organization for 0–3 years (0.60 ± 1.48) vs. 10–19 years (3.50 ± 4.31), (ii) were early- (1.96 ± 2.29) or mid-career investigators (3.38 ± 3.53) vs. late-career investigators (8.10 ± 5.74), and (iii) were mid- (3.38 ± 3.53) or late-career investigators (8.10 ± 5.74) vs. staff (0.55 ± 1.50). Differences in the mean number of formal mentors, both within and outside the Center, were not significant by any respondent characteristic. For informal mentorship, the mean number of informal mentors within the Center was significantly different between investigators (4.94 ± 3.59) and staff (3.59 ± 3.72). Differences in the mean number of informal mentors within the Center were not significant between sites, years at the organization, or career stages.

**Table 2:** Numbers of mentors or mentees, using two-sample *t*-tests for comparing two groups and analyses of variance for comparing more than two groups.

Mentorship type	Formal mentorship				Informal mentorship
Respondent characteristic	Number of mentors	Number of mentors within the center	Number of mentors outside the center	Number of mentees	Number of mentors within the center
Mean ± SD overall	1.39 ± 1.86	2.20 ± 1.38	0.58 ± 1.19	1.75 ± 3.14	4.18 ± 3.70
Mean ± SD by role at the center					
Investigator	1.60 ± 1.74	2.23 ± 1.30	0.69 ± 0.96	3.31 ± 3.96	4.94 ± 3.59
Staff	1.24 ± 1.96	2.21 ± 1.47	0.49 ± 1.20	0.55 ± 1.52	3.59 ± 3.72
Difference in means (*p*-value)	0.30	0.94	0.42	0.15	0.05* [Cohen's *d* = 2.00]
Mean ± SD by site					
A	1.41 ± 1.69	2.20 ± 1.38	0.40 ± 0.84	1.94 ± 3.16	4.65 ± 3.62
B	1.38 ± 2.00	2.23 ± 1.41	0.75 ± 1.27	1.59 ± 3.14	3.78 ± 3.74
Difference in means (*p*-value)	0.93	0.94	0.10	0.55	0.20
Mean ± SD by years at the organization					
0–3	1.65 ± 1.76	2.08 ± 1.29	0.56 ± 1.22	0.60 ± 1.48	3.93 ± 3.83
4–9	1.00 ± 1.78	2.33 ± 1.50	0.68 ± 2.62	1.63 ± 0.48	4.12 ± 3.79
10–19	1.39 ± 1.78	2.40 ± 1.34	0.53 ± 0.96	3.50 ± 4.31	5.30 ± 3.66
20+	1.00 ± 1.41	1.50 ± 0.57	0.33 ± 1.00	3.11 ± 4.13	2.67 ± 2.64
Difference in means (*p*-value)	0.38	0.67	0.87	0.00*** [*η*^2^ = 0.25]	0.23
Mean ± SD by career stage					
Early-career investigator	1.59 ± 1.82	2.08 ± 1.15	0.67 ± 1.05	1.96 ± 2.29	4.63 ± 3.88
Mid-career investigator	1.62 ± 1.75	2.80 ± 2.05	0.75 ± 0.77	3.38 ± 3.53	5.56 ± 3.77
Late-career investigator	1.75 ± 1.66	2.00 ± 0.82	0.75 ± 1.16	8.10 ± 5.74	4.75 ± 2.49
Staff	1.22 ± 1.93	2.21 ± 1.47	0.49 ± 1.18	0.55 ± 1.50	3.61 ± 3.67
Difference in means (*p*-value)	0.70	0.78	0.78	0.00*** [η^2^ = 0.57]	0.22

* *p* < 0.05; **p < 0.01; ***p < 0.001.

### 3.2 Satisfaction with mentoring

The mean satisfaction with mentoring (higher indicates more satisfied) was 5.98 ± 2.48. There was a statistically significant difference in mean satisfaction (*p* = 0.002) between investigators (6.63 ± 2.41) and staff (5.25 ± 2.37). Differences in mean satisfaction between sites, years at the organization, or career stages were not significant.

### 3.3 Barriers to mentorship

Figures 1a,b show the frequency of responses to the following two questions, respectively: “What factors, if any, have limited you finding a mentor at [the Center]?” and “What factors, if any, have limited you becoming a formal mentor?”

For investigators, “None” (i.e., no limiting factors experienced) was selected more often than any other response option for both questions. Common limiting factors for not finding a formal mentor were “Nobody seems to have the time to mentor” and “I haven't been able to find a good match for my interests.” Common limiting factors to becoming a formal mentor were “No one has contacted me about becoming a mentor” and “I do not currently have time to be a mentor.”

For staff, common limiting factors for not finding a formal mentor were “I don't even know where to start looking for a mentor” and “I don't believe I need a mentor/Finding a mentor has not been a priority for me.” Common limiting factors to becoming a formal mentor were “No one has contacted me about becoming a mentor” and “I am not confident in my ability to be a mentor.”

### 3.4 Network analysis

**Figure 2:**
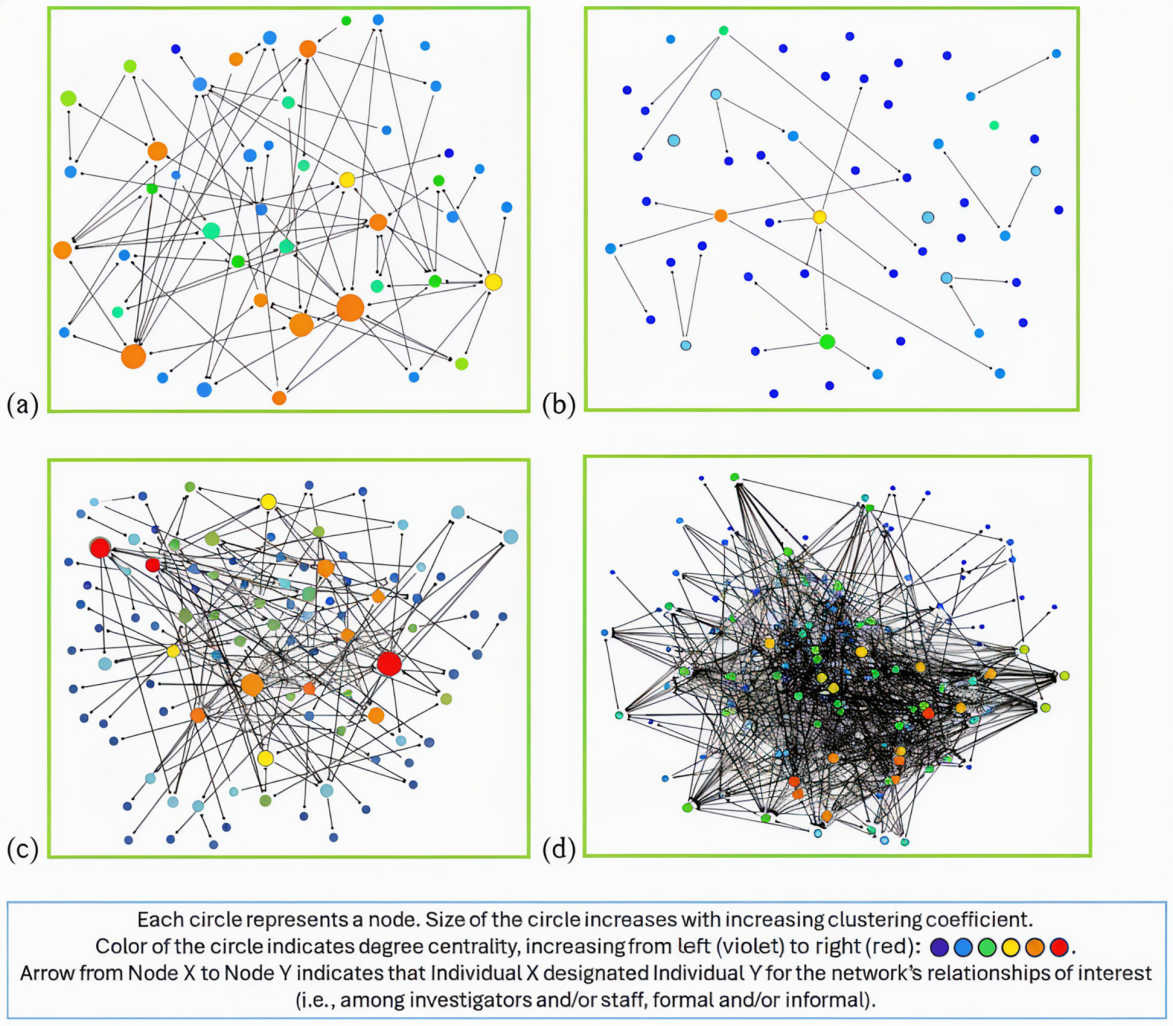
Visual maps for **(a)** network 1 (investigators, formal mentorship), **(b)** network 2 (staff, formal mentorship), **(c)** network 3 (investigators and staff, formal mentorship), and **(d)** network 4 (investigators and staff, formal and informal mentorship).

Figures 2a–d show the visual maps for Networks 1 through 4, respectively. For each network's visual map, node size is proportional to the associated individual's clustering coefficient, and an edge drawn as an arrow from Node X to Node Y indicates that Individual X designated Individual Y for the network's relationships of interest (i.e., among investigators and/or staff, formal and/or informal). Figure 2a shows notably more edges between nodes than Figure 2b, suggesting that there are considerably more formal mentoring relationships among investigators than among staff. Figure 2c shows notably more edges between nodes than Figure 2d, suggesting that informal mentoring relationships substantially contribute to the total number of mentoring relationships.

The findings that are visually depicted in Figure 2 are mirrored in Table 3, which compares the network metrics between Networks 1 and 2 (of investigators and staff, respectively) and between Networks 3 and 4 (of both investigators and staff). Differences in means were significant for Networks 1 and 2 in degree centrality and betweenness centrality. That is, investigators had more formal mentorship connections with other investigators than staff had with other staff, and investigators more than staff served as bridges between members in their respective formal mentorship networks. Differences in means were significant for Networks 3 and 4 in mean distance and degree centrality. In other words, formal and informal mentorship together, compared to formal mentorship alone, enabled smaller degrees of separation between members, as well as more connections to other members, in the combined investigator-staff network.

**Table 3:** Network metrics; for network density, proportion and difference in proportions (*p*-value), using (i) chi-square test to compare networks 1 and 2 and (ii) McNemar's test to compare networks 3 and 4; for other metrics, mean ± SD and difference in means (*p*-value), using (i) two-sample *t*-tests to compare networks 1 and 2 and (ii) paired-sample *t*-tests to compare networks 3 and 4.

Metric	Network 1 (investigators, formal mentorship)	Network 2 (staff, formal mentorship)	Network 3 (investigators and staff, formal mentorship)	Network 4 (investigators and staff, formal and informal mentorship)
Network density	0.09	0.02	0.14	0.28
	[*p* = 0.00***; (*φ* = 0.37)]		(*p* = 1.00)	
Mean distance	3.50 ± 8.94	1.31 ± 3.75	3.41 ± 0.35	1.32 ± 0.50
	(*p* = 0.34)		[*p* = 0.00***; (Cohen's *d* = 0.21)]	
Clustering coefficient	0.09 ± 1.57	0.00 ± 0.00	0.04 ± 1.63	0.09 ± 2.57
	(*p* = 0.82)		(*p* = 0.91)	
Degree centrality	0.06 ± 0.00	0.01 ± 0.00	0.02 ± 0.00	0.01 ± 0.00
	[*p* = 0.00***; (Cohen's *d* = 0.23)]		[*p* = 0.00***; (Cohen's *d* = 0.13)]	
Betweenness centrality	0.04 ± 0.00	0.00 ± 0.00	0.01 ± 0.00	0.01 ± 0.00
	[*p* = 0.00***; (Cohen's *d* = 0.12)]		(*p* = 1.00)	

*p < 0.05; **p < 0.01; ***p < 0.001.

For both Networks 3 and 4, satisfaction with mentoring was highly correlated (quantified in parentheses for Networks 3 and 4, respectively) with mean distance (*r* = −0.955, *p* < 0.0001; *r* = −0.898, *p* < 0.0001), clustering coefficient (*r* = 0.965, *p* < 0.0001; *r* = 0.998, *p* < 0.0001), and degree centrality (*r* = 0.990, *p* < 0.0001; *r* = 0.989, *p* < 0.0001). In other words, respondents who had greater satisfaction with mentoring infrastructure were those who had smaller degrees of separation from other members, more connections with members who were also connected with each other, and more connections with other members. For Network 4 of both formal and informal mentorship, members' satisfaction was also strongly positively correlated with their serving as bridges between other members (*r* = 0.996, *p* < 0.0001), whereas these values were negatively correlated for Network 3 of only formal mentorship (*r* = −0.660, *p* < 0.0001).

## 4 Discussion

In this project, we examined mentorship experiences of investigators and staff at a federally funded health research center. We used a cross-sectional survey to inquire about their formal and informal mentoring connections, satisfaction with mentorship, and reasons for mentoring-related challenges. Findings indicated that a larger proportion of investigators had at least one mentor, at least one mentor within the Center, and at least one mentee, while staff had a larger average number of mentors outside the Center. Satisfaction with mentorship was higher for investigators than for staff, and an analysis of formal and informal mentorship networks showed that the mean number of formal mentees was significantly different for respondents who (i) had been at the organization for 0–3 years vs. 10–19 years, (ii) were early- or mid-career investigators vs. late-career investigators, and (iii) were mid- or late-career investigators vs. staff. The mean number of informal mentors within the Center was significantly higher for investigators than for staff. Across all of the examined networks (investigators, formal mentorship; staff, formal mentorship; investigators and staff, formal mentorship; and investigators and staff, formal and informal mentorship), we found that, in terms of mentoring relationships, (i) network members are sparsely interconnected relative to the maximum level of interconnectedness possible (low network density), (ii) networks are highly decentralized (low clustering coefficient), (iii) network members are generally connected to a small proportion of other members (low degree centrality), and (iv) network members have large degrees of separation from most other members (low betweenness centrality).

For investigators, a commonly perceived barrier to both receiving and providing mentorship was limited time availability of the individuals who could fulfill the mentor role. Another common barrier to providing mentorship was not being asked to serve as a mentor, suggesting that there may be an opportunity to formally implement a process to assess mentorship needs and availability to identify matches that might not be apparent otherwise. Mentor-mentee matching processes have been gaining increased attention in many non-research oriented entities (18, 19), and research organizations may benefit from adapting such processes for their use.

Although mentoring relationships in research are formalized for research career training initiatives, including fellowships (20, 21), less is known about the extent to which such relationships are common and expected of individuals in other career tracks (e.g., staff) within the research work environment. Common staff-reported barriers to mentoring relationships were not knowing how to start the process of looking for a mentor and not feeling confident to serve as a mentor. These hurdles may be addressable through strategies identified by a 2024 global evidence synthesis aimed at improving research mentorship in resource-limited settings (22). Two of the strategies may be particularly relevant—(i) encouraging peer mentorship, which provides exposure to mentorship without the pressure of guiding a junior colleague's career and (ii) nurturing a culture where mentees are expected to serve as mentors to others, so that such expectations could help build a cascade of mentorship over time. Another promising strategy is a structured mentoring program published in 2023 that specifically focuses on research staff in a university setting (23). The program encompasses mentor training activities, explicit appointment of experienced staff to facilitate creation of mentor-mentee matches, and non-binding permission for the mentor and mentee to each reassess their matches and dissolve the mentoring relationship if the match is not meeting needs. Each of these aspects of the program could contribute to increasing the confidence of staff in getting involved with mentorship.

Our network analysis found that satisfaction with mentoring is correlated with more and closer connections to other closely connected network members, and in the case of the combined formal and informal mentorship network, also with serving as a bridge between other members. Thus, potential approaches to foster additional connections among investigators and staff within an organization can draw on strategies with strong evidence from the fields of organizational management and operations research. These strategies that could contribute to strengthened relationships among co-workers include creating forums for open communication between multiple levels of the organization (24, 25) and opportunities for collaborative problem-solving that call for innovative solutions to be devised as a multi-perspective team (e.g., efforts to improve work processes that bring together colleagues in various organizational roles and get them to together undergo experiential learning) (26). Feeling connected to co-workers is crucial for employees' motivation and well-being (27, 28), which could in turn fuel their willingness to take part in new initiatives for improved mentoring practices stemming from this work. Warranting further investigation is our finding that, for the formal mentorship network, satisfaction with mentoring was negatively correlated with serving as a bridge between other members. This finding may stem from network members’ experiences of high effort associated with formal mentorship (e.g., required mentorship meetings even when there are many other demands on their time) (29, 30).

Although our project's findings suggest that the abovementioned strategies may be beneficial to improve mentorship experiences of investigators and staff, an important limitation of our work is its cross-sectional focus on one research organization. However, our goal in sharing this work is to communicate not only our findings but also the survey-based approach that we took to examine mentorship practices, which can be adapted and applied by other research organizations seeking to assess their own employees' mentorship experiences. Six additional limitations are also worth noting. First, we included data from respondents who did not answer every survey question. Even though there were no noticeable patterns that suggest that the missing data were not at random, there may be characteristics of respondents that we did not collect that impacted which questions were skipped by whom. Second, findings may be subject to researcher bias, as we may have interpreted improvement opportunities to be addressable by strategies related to ones that we know to have been considered for implementation by the participating research organization. To demonstrate the strategies' actual impact, further work is needed to assess change over time resulting from implementing one or more of those strategies. Third, we conducted multiple statistical comparisons across subsets of our data. Although this may increase the likelihood that the statistically significant relationships that we found are due to chance, we did not adjust the significance level accordingly when reporting our findings, given the primary focus of this work to explore potential relationships between variables to generate hypotheses that can be more formally tested in subsequent work. Fourth, although we report on how satisfaction with mentoring is correlated with the network metrics, this project did not include examining how mentoring practices differ between more and less connected network members. Further work to examine such differences may help identify additional recommended mentoring practices for consideration by research organizations. Fifth, our survey methodology did not include formally (i) conducting pilot testing of the developed survey prior to full deployment, (ii) analyzing open-ended responses that were collected as part of the survey, or (iii) being guided by social learning theories or organizational mentorship models in designing the survey [although our work did build on how mentoring relationships have been viewed as social networks (15)]. Formal incorporation of such methodological steps may have enabled a higher-quality survey instrument, as well as a deeper understanding of both the reasons behind our findings and how they explicitly compare to relevant conceptual theories and models. Sixth, because the investigators and staff who were invited to participate in the survey were based on Center-wide email lists at the time of survey administration (rather than on the organization's personnel records), we did not collect data on non-respondents' role at the Center, site, years at the organization, or career stage. This lack of data hinders our ability to accurately gauge the extent to which the different member groups are represented by the survey participants.

This work adds to extant literature by examining mentoring experiences of both investigator and staff in a research organization, highlighting differences between these employee groups in their engagement with mentoring and reported barriers to mentoring involvement. Both our survey-based approach and our findings are especially timely given the increased attention that mentorship is receiving as a critical requirement for successful careers, motivated employees, and workplace productivity. Future work is needed to delve deeper into similar and unique needs that research organizations have for mentoring compared to other types of organizations, as well as to develop a firmer understanding of how best to measure and address equitable improvement of mentoring experiences for employees from diverse backgrounds.

## Data Availability

The original contributions presented in the study are included in the article, and further inquiries can be directed to the corresponding author.
